# Case Report: A Case of Locally Advanced Pancreatic Cancer Which Achieved Progression Free for Over 12 Months by Subsequent Therapy with Anlotinib Hydrochloride Plus Tegafur-Gimeracil-Oteracil Potassium (TS-1)

**DOI:** 10.3389/fonc.2022.862600

**Published:** 2022-07-01

**Authors:** Dongcheng Luo, Sina Liao, Qian Li, Youzhi Lin, Junbao Wei, Yongqiang Li, Xiaoli Liao

**Affiliations:** ^1^ Department of First Chemotherapy, Guangxi Medical University Cancer Hospital, Nanning, China; ^2^ Hepatobiliary Surgery Department, Guangxi Medical University Cancer Hospital, Nanning, China; ^3^ Radiotherapy Department, Guangxi Medical University Cancer Hospital, Nanning, China

**Keywords:** advanced pancreatic cancer, anlotinib, TS-1, gimeracil and oteracil potassium capsules, targeted therapy, chemotherapy

## Abstract

Titled the “most destructive of all cancers”, pancreatic cancer is a malignant tumor with a very poor prognosis and has a poor response to systemic therapy. At present, several studies have shown that tegafur-gimeracil-oteracil potassium (hereinafter referred to as TS-1) is no less superior to gemcitabine in the treatment of advanced pancreatic cancer. In addition, a number of current clinical studies have shown that targeted therapy combined with chemotherapy reflects therapeutic advantages in pancreatic cancer. Moreover, *in vitro* and *in vivo* experiments have also demonstrated that anlotinib can curb the proliferation of pancreatic cancer cells and induce their apoptosis. Here, we report for the first time that a patient with locally advanced pancreatic cancer achieved good efficacy after switching to TS-1 chemotherapy combined with anlotinib targeted therapy. Previously, the disease of the patient still rapidly progressed without control following the first switch to abraxane combined with gemcitabine chemotherapy (AG regimen) due to the progression after chemo-radiotherapy. In this case, the patient achieved progression-free survival (PFS) of over 14 months *via* the treatment with anlotinib targeted therapy combined with TS-1 chemotherapy and secondary radiotherapy (prior to secondary radiotherapy, the patient achieved a PFS of nearly 12 months *via* the treatment with oral anlotinib combined with TS-1). Up to now, the progress of the disease is ceased. The oral administration of targeted therapy and chemotherapy are still in progress and the general condition of the patient is good. This suggests that patients with advanced pancreatic cancer may benefit from treatment with the anlotinib targeted therapy combined with TS-1 chemotherapy.

## Background

Pancreatic cancer is the seventh leading cause of cancer-related death worldwide. In 2018, there were 432,242 deaths across the world due to pancreatic cancer (1). The 5-year survival rate for pancreatic cancer is less than 10% (2). One of the reasons is that pancreatic cancer has an insidious onset. It is often diagnosed at an advanced stage and has little chance for radical surgery. Furthermore, the peculiar biological characteristics of pancreatic cancer lead to the tolerance of chemotherapy and radiotherapy (3), ultimately resulting in a poor prognosis in patients with pancreatic cancer. Therefore, it is imperative to explore treatment options that may have good efficacy for patients with pancreatic cancer. This study reports a case of locally advanced pancreatic cancer, in which the treatment regimen after the progression with chemo-radiotherapy was changed to abraxane combined with gemcitabine (AG regimen) chemotherapy. However, the disease still progressed rapidly and was out of control. The regimen was then switched to TS-1 chemotherapy combined with anlotinib targeted therapy, by which good efficacy was obtained.

## Case Description

**Figure 1:**
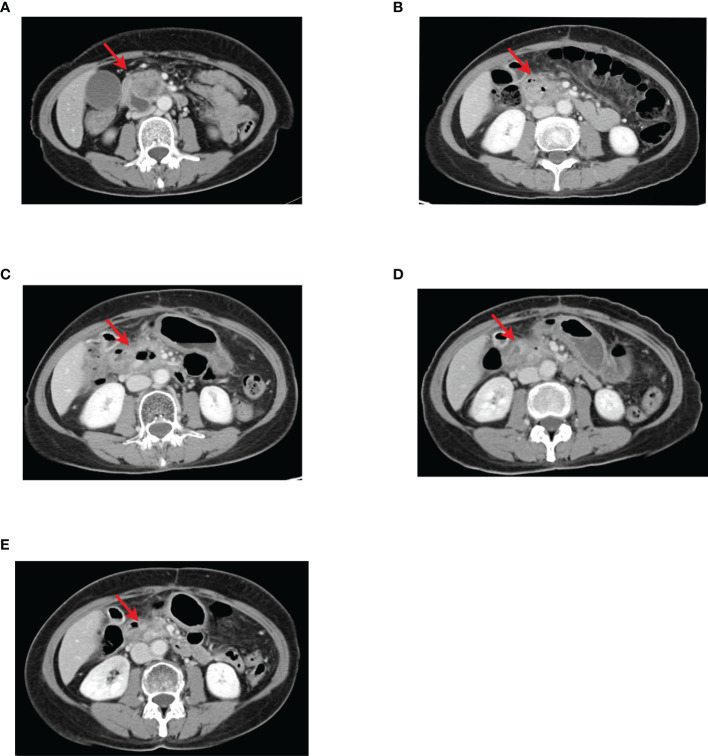
CT images of pancreatic lesions during concurrent chemo-radiotherapy followed by sequential chemotherapy, with red arrows indicating the location of pancreatic head lesions. **(A)** The initial CT examination revealed a mass in the head of the pancreas, about 3.6 × 3.5 cm. **(B)** After concurrent chemo-radiotherapy, the pancreatic head cancer lesion did not change much compared with the previous, about 3.9 × 3.3 cm. **(C–E)** One to six cycles of sequential chemotherapy with gemcitabine + TS-1 gradually reduced the pancreatic head lesion and the efficacy evaluation was PR.

The patient is female and 58 years old, with a height 150 cm, weight 46.5 kg, and body surface area 1.4188 m^2^. The patient visited Guangxi Medical University Affiliated Tumor Hospital on April 17, 2019, due to generalized weakness for more than 1 month as well as icteric skin and sclera for 1 week. The results of her physical examination revealed that she had mild yellowish discoloration of the skin mucosa and sclera all over the body, as well as mild epigastric tenderness without rebound tenderness. The tumor markers on admission to hospital were 323.5 U/Ml for CA19-9, 69 U/Ml for CA125, and 7.08 ng/Ml for CEA, with CA19-9 significantly increased. Total bilirubin was 47.4 umol/L, direct bilirubin was 36 umol/L, and indirect bilirubin was 11.4 umol/L. Total and direct bilirubin were mildly elevated and ALT was 302 U/L. The results of the MRI + MRCP on liver showed: 1. A pancreatic head mass was noted with low level biliary obstruction, a possible pancreatic cancer was considered. 2. Small cysts were visible in the left liver lobe, and gallbladder signal changed. A possible cholestasis was considered. The results of the upper abdominal CT showed: There was a mass shadow in the head of the pancreas, which lacked blood supply, about 3.6 cm × 3.5 cm in size. The pancreaticobiliary duct was significantly dilated with “double-tube sign”. The mass contacted duodenum and superior mesenteric vein, while the direct invasion was inapparent radiologically. The demarcation with the superior mesenteric artery and vein was blurred. Combined with imaging information and clinical manifestations, the comprehensive determination is as follows: 1. Significantly enlarged mass shadow in the head of the pancreas and the dilated pancreaticobiliary duct suggested pancreatic head cancer; 2. No distant metastasis was developed in the lung; 3. The blurred demarcation between the mass of the pancreas head and the superior mesenteric artery and vein indicated local advanced stage (**Figure 1A**). Hence, the disease was considered initially as a pancreatic cancer.

**Figure 2:**
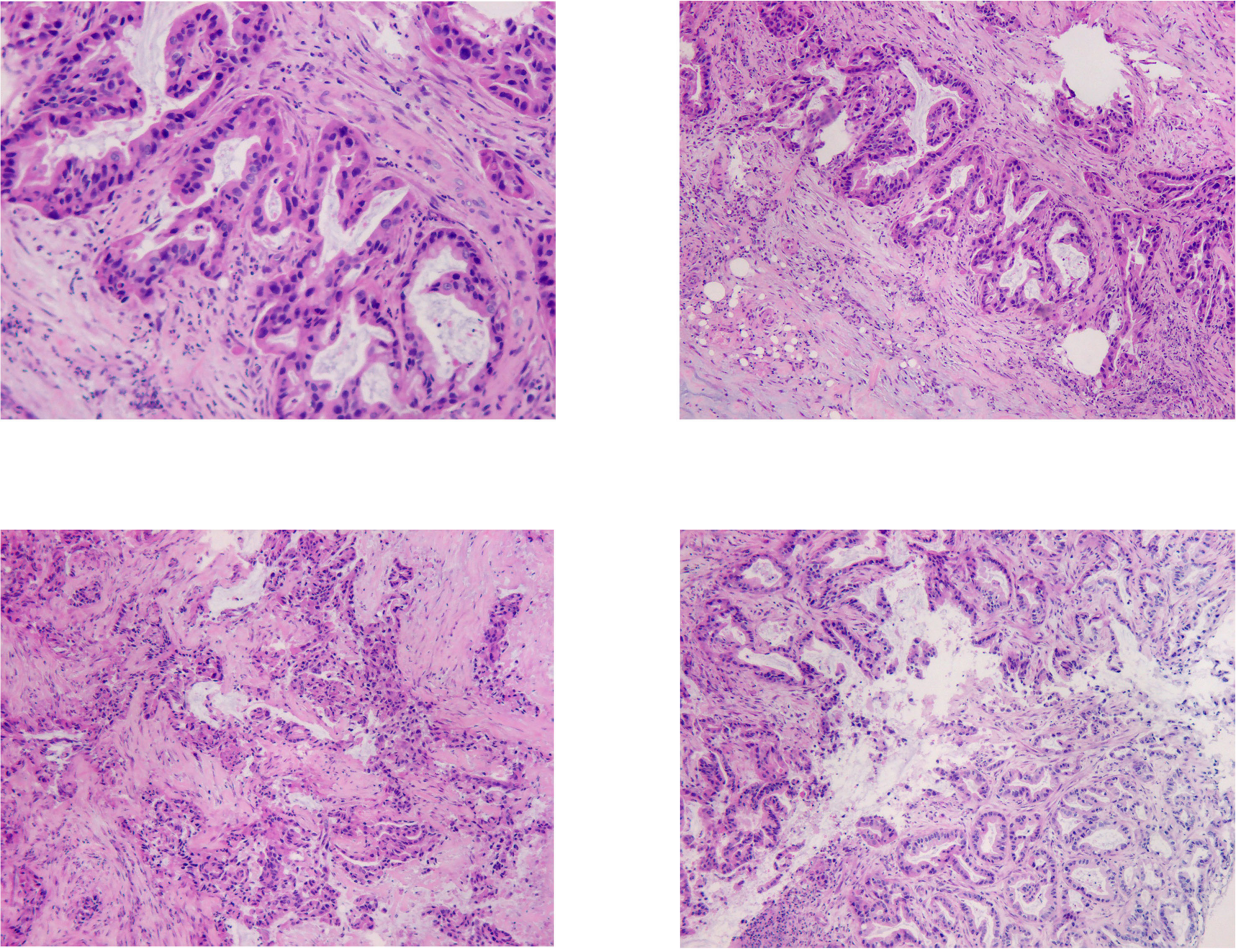
In the HE staining pictures, the tissue at the upper edge of the pancreas was shown, no normal pancreatic structure was seen, and irregular carcinomatous glandular infiltration was observed. Glandular epithelial cells were enlarged, varying in size, crowded, and disorganized. Nuclei were enlarged and irregular, chromatin was thickened. Stroma was present, accompanied by significant fibrogenic response.

For further diagnosis, exploratory laparotomy was performed for the patient after general anesthesia on April 24, 2019. It was shown intraoperatively that: A mass of about 3.5 cm × 3.5 cm × 2 cm in the head of the pancreas invaded the superior mesenteric artery and vein, and the mesentery root contracted because of being invaded. Multiple lymph nodes were enlarged and hardened in Group 13 posterior to the pancreatic head of the abdominal cavity and Group 8 at the upper edge of the pancreas. Subsequently, a gray and white hard tissue at the upper edge of the pancreas was resected for intraoperative rapid pathological examination. The pathological findings revealed invasive or metastatic adenocarcinoma in the fibrous tissue (lymph nodes at the upper edge of the pancreas, **Figure 2**). The tumor was in the locally advanced stage, which could not be resected radically. Finally, Roux-en-Y Cholecystojejunostomy, abdominal lymph node biopsy as well as abdominal adhesiolysis were performed. Conclusively, combined with the patient’s medical history, laboratory test (CA19-9 significantly increased), imaging examination (pancreatic head mass shadow lacked blood supply, and pancreaticobiliary duct significantly dilated, presenting “double tube sign”), pathological findings (**Figure 2**), the disease of the patient was diagnosed as pancreatic head adenocarcinoma with abdominal lymph node metastasis (pT4N2M0, stage III), which was confirmed after palliative surgery.

Following the diagnosis, TS-1 chemotherapy combined with concurrent three-dimensional conformal radiotherapy for pancreatic head lesions was initiated on May 27, 2019. The details of the chemotherapy dose are: TS-1 60 mg in the morning and 40 mg in the evening po d1-14, Q3w. The radiotherapy target area is GTV, with the residual lesion after pancreatic surgery visible by imaging, and the PGTV, expanded from GTV by 0.5 cm. The prescribed dose of radiotherapy is: 6MV-X-ray, IMRT, PGTV: 50 Gy/25f, and the organs at risk of the disease were given limited doses. The course of concurrent chemo-radiotherapy was uneventful. The results of repeat abdominal CT on August 12, 2019 showed that the pancreatic head cancer lesion, about 3.9 cm× 3.3 cm, did not change much compared with the previous (**Figure 1B**), suggesting stable disease (SD). From August 14, 2019, to January 20, 2020, the patient was given sequential chemotherapy with gemcitabine + TS-1, lasting for the first to sixth cycles, with the dose of gemcitabine: 1300 mg ivdrip d1, d8 + TS-1 40 mg po bid, d1-d14, Q3W. After two cycles, the results of the repeated abdominal CT on October 22, 2019 showed that the pancreatic head cancer lesion reduced the size to approximate 2.3 cm × 2.1 cm. And the results of the repeated abdominal CT on December 11, 2019, after four cycles, showed that the pancreatic head cancer lesion had diminished to 1.5 cm × 1.7 cm. Then, regular follow-up started after the end of the six cycles of chemotherapy. The results of abdominal CT in the follow-up period on March 14, 2020, showed that the pancreatic head cancer lesion had decreased to the size of approximate 1.1 cm × 0.9 cm (**Figures 1C–E**). The efficacy of the Gemcitabine + TS-1 regimen in the treatment was partial response (PR).

**Figure 3:**
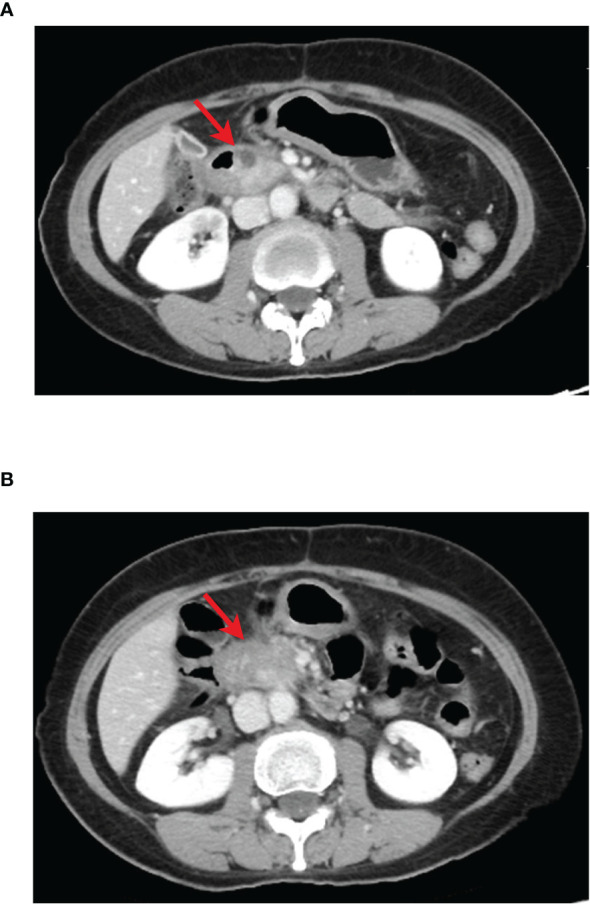
CT images of pancreatic cancer lesion during first-line treatment, red arrow indicates pancreatic lesion. **(A)** The results of CT reexamination 3 months after the end of sequential chemotherapy showed a slightly enlarged pancreas head mass, prior to AG regimen treatment. **(B)** The results of CT after two cycles of AG regimen treatment showed significantly enlarged pancreatic head mass compared with that before treatment.

The results of abdominal CT in follow-up on June 29, 2020, showed that the pancreatic head mass was larger than the previous, about 1.0 cm × 2.3 cm (**Figure 3A**). And the BRCA1/2 gene test results on July 30, 2020, showed no mutation was detected. Immunohistochemically, MLH1 (+), PMS2 (+), MSH2 (+), and MSH6 (+), suggested pMMR, which indicated no signs for subsequent use of olaparib and immune checkpoint inhibitors. On August 12, 2020, and September 4, 2020, the patient received Cycle 1-2 first-line treatment with gemcitabine + abraxane regimen at the following doses: gemcitabine 1000 mg ivdrip d1, d8 + abraxane 150 mg, ivdrip d1, d8; Q3w.

**Figure 4:**
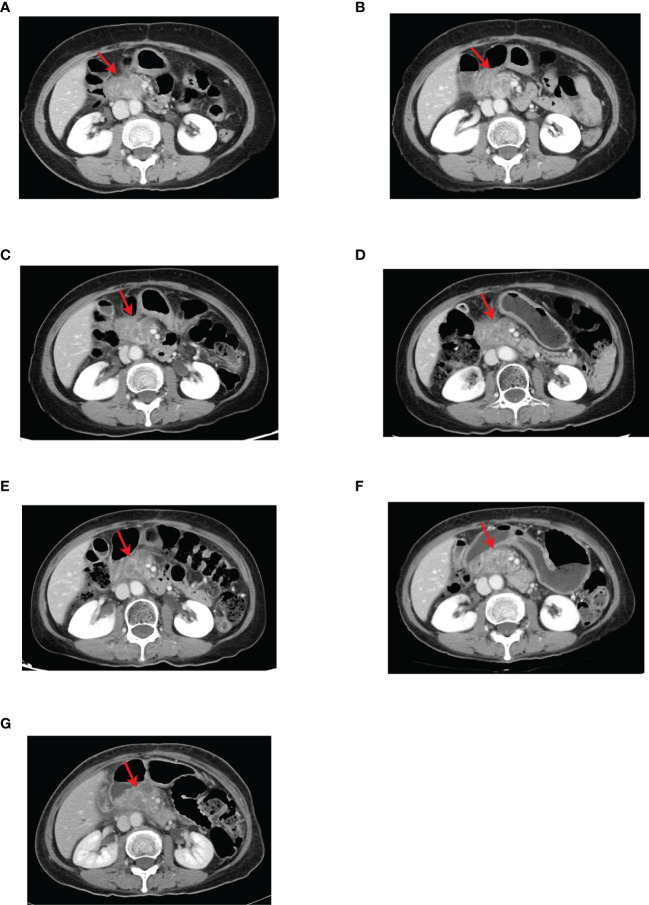
CT images of the pancreatic head lesion in the second-line course with red arrows indicating the location of the pancreatic head lesion. **(A)** Before anlotinib-targeted therapy combined with TS-1, the tumor size was about 2.4 cm × 4.3 cm. **(B)** CT after three cycles of anlotinib targeted therapy combined with TS-1 showed that the pancreatic head tumor was reduced to approximate 2.5 cm × 3.3 cm. **(C)** CT after eight cycles of anlotinib targeted therapy combined with TS-1 showed that the pancreatic head tumor was slightly enlarged compared with that after three cycles of treatment, about 4.2 cm × 3.1 cm. **(D)** CT showed no significant change in the size of the pancreatic head tumor after adjusting the anlotinib dose for two cycles. **(E)** After three cycles and **(F)** five cycles, CT showed that the pancreatic head tumor was slightly larger than the previous, about 4.3 cm × 3.1 cm. **(G)** CT image after adjusting the dose of anlotinib for six cycles showed no significant change in the size of the pancreatic head tumor compared with the previous, and the efficacy was evaluated as SD.

The results of CT examination in follow-up on October 12, 2020, showed that the pancreatic head mass was significantly larger than before, about 2.4 cm × 4.3 cm (**Figures 3B**, **4A**), and then second-line treatment was initiated. After consultation and discussion within the Department, it was suggested that the patient should receive anlotinib targeted therapy combined with TS-1 chemotherapy. Then the patient was administered TS-1 + anlotinib for treatment lasting Cycle 1-8 from October 14, 2020, to April 3, 2021, with detailed dosis of TS-1 40 mg bid po d1-d14, anlotinib 8 mg qd po d1-d14, q3w. After three cycles, the results of CT reexamination on December 29, 2020 showed that the pancreatic head mass had reduced size to 2.5 cm × 3.3 cm (**Figure 4B**). The results of the CT examination in follow-up on April 20, 2021, after eight cycles showed: Pancreatic head mass was slightly enlarged, about 4.2 cm × 3.1 cm (**Figure 4C**).

**Figure 5:**
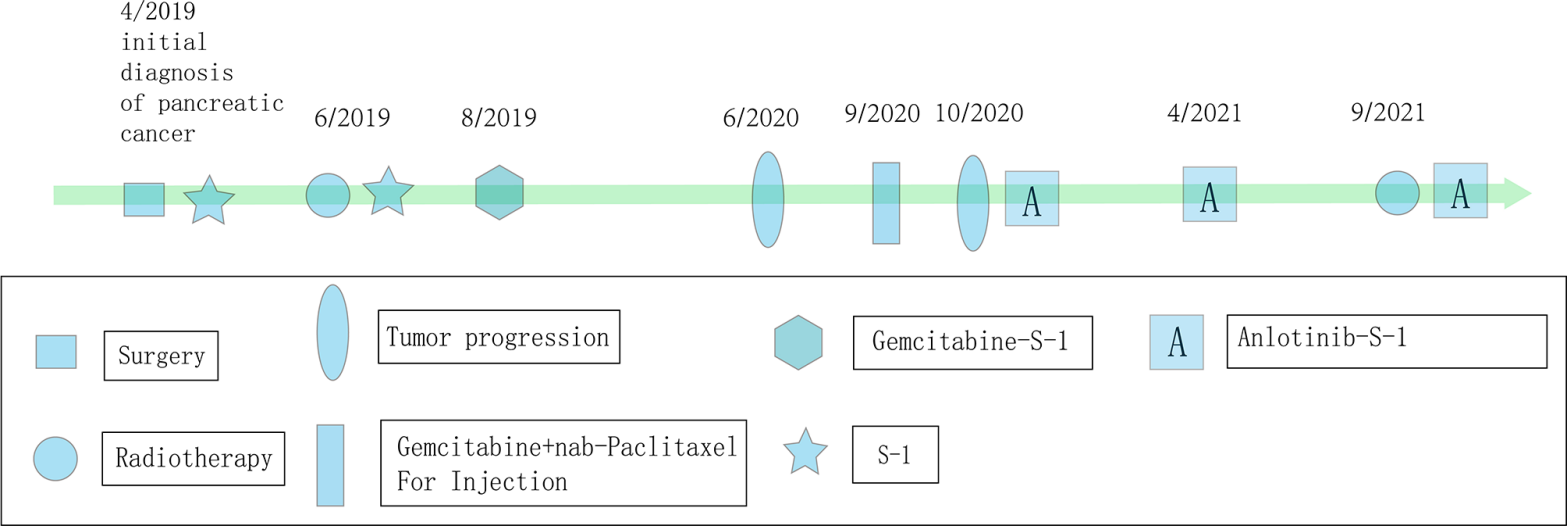
Flowchart of the patient’s treatment history.

**Figure 6:**
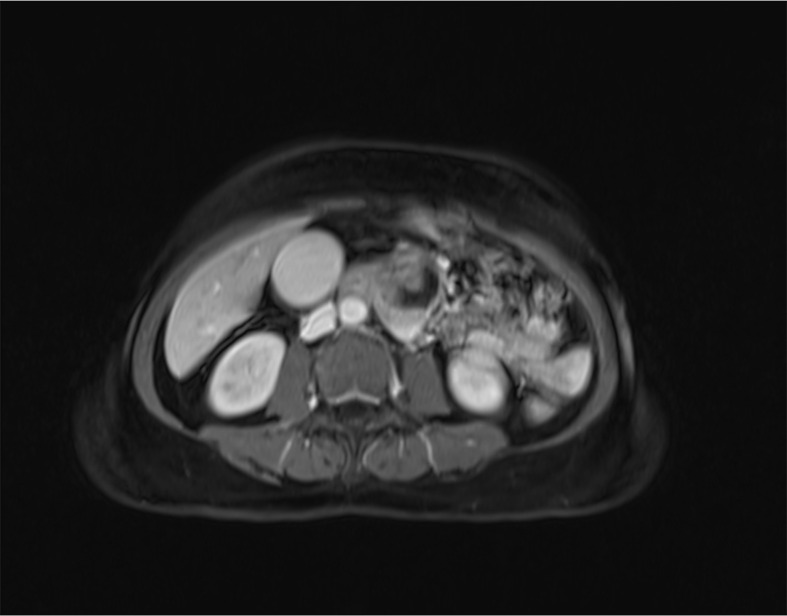
MRCP images before treatment.

The results of the CT examination in follow-up showed that the pancreatic head tumor was stable and tended to enlarge. In view of the good control of other lesions and the smooth process of targeted therapy and chemotherapy without significant adverse reactions, the dose of anlotinib was adjusted to 10 mg. From April 23, 2021, to September 15, 2021, the patient was treated with TS-1 + anlotinib for six cycles with the detailed doses of TS-1 40 mg bid po d1-d14, anlotinib 10 mg qd po d1-d14, q3w. During this period, the results of CT reexaminations for three times showed that the pancreatic head mass was overall stable (**Figures 4D–F**), and the signs of local radiotherapy was observed and evaluated by the radiotherapy department. Therefore, the secondary radiotherapy was performed from September 26, 2021, to November 3, 2021, with radiotherapy prescription of 6MV-X-ray, IMRT, PGTV1: 45 Gy/25f, PGTV2: 42.5 Gy/25f. After radiotherapy, the results of the CT reexamination on November 5, 2021, showed that the pancreatic head mass did not change much compared with the previous, about 4.3 cm × 3.1 cm (**Figure 4G**). Up to now, the patient’s tumor status remains stable, with a size of 4.3 cm × 3.1 cm. And the patient’s general condition is good, without abdominal pain, jaundice, abdominal fullness discomfort, lack of appetite, indigestion, nausea, vomiting, hematemesis, melena, or symptoms. The patient has a performance score (ECOG scale) of 1 and NRS pain score of 0. Now, the patient continued to receive treatment with anlotinib combined with TS-1. Its adverse reactions (first-degree hypertension, Grade I hand-foot syndrome) were well monitored *via* regular administration of nifedipine sustained-release tablets and skin moisturizing care. The patient had no diabetes and other chronic diseases, infectious diseases, endemic diseases, or venereal diseases. The patient reported that she recovered well and could maintain normal daily activities. The patient’s treatment history is shown in **Figure 5**. The MCPR image of the patient before treatment is shown in **Figure 6** as a reference.

## Discussion

In this case, we describe a patient with locally advanced pancreatic cancer (pT4N2M0, stage III). This patient has been treated with anlotinib targeted therapy combined with TS-1 chemotherapy since October 2020 with a good outcome for 14 months. During this period, the pancreatic head cancer lesion was well monitored, once reduced in size, it remained stable on subsequent treatment. No distant metastasis was found. The treatment reduced the tumor burden, addressed the clinical symptoms, had less side effects of drugs, improved the quality of life (QoL), and prolonged the survival of the patient. To the best of our knowledge, it is the first report that anlotinib combined with TS-1 achieved a good efficacy in the treatment of advanced pancreatic cancer.

In this case, following the patient with pancreatic cancer was evaluated as unresectable locally advanced pancreatic cancer *via* the exploratory laparotomy and received concurrent chemo-radiotherapy, the patient entered the sequential chemotherapy phase of gemcitabine combined with TS-1 chemotherapy regimen (GS regimen) in line with the *Guidelines of Chinese Society of Clinical Oncology (CSCO) for Pancreatic Cancer 2019.* The study results of GEST, a phase III clinical trial, shows gemcitabine combined with TS-1 chemotherapy regimen (GS regimen) has a significant advantage in progression-free survival (PFS) and response rate (RR) (4). The GS regimen played a certain role in the tumor control of this patient, but the tumor began rapid progression after the end of six cycles of chemotherapy. At that time, there was no existing guideline basis recommending the use of targeted drugs for coping with the results of genetic testing. As recommended by the NCCN guidelines, treatment can be continued with a regimen of gemcitabine +albumin-bound paclitaxel for patients with patent biliary tracts and good nutritional status (5) (NCCN Guidelines, Pancreatic Adenocarcinoma, Version 1, 2022). Therefore, we changed the regimen to gemcitabine combined with abraxane (AG regimen). This method significantly prolonged the median survival (mPFS = 5.5 months; mOS = 8.7 months) of treatment-naive patients with metastatic pancreatic cancer compared with gemcitabine monotherapy in the MPACT phase III clinical study, so it was also selected as the first-line treatment option (6). However, after two cycles of this regimen, rapid tumor progression was still evident. Therefore, we considered that the patient had developed resistance to gemcitabine at the time.

Although gemcitabine is the drug of choice for first-line treatment of metastatic pancreatic cancer, an increasing number of studies have also revealed the resistance and limitations of gemcitabine in the treatment of pancreatic cancer. Gemcitabine resistance remains an insurmountable difficulty in the systemic treatment of pancreatic cancer.

According to the NCCN Guidelines for Pancreatic Adenocarcinoma (Version 2, 2021) (7), fluorouracil-based regimens can be considered for second-line treatment of pancreatic cancer after failure of gemcitabine + albumin-bound paclitaxel chemotherapy regimens. The NCCN recommended second-line chemotherapy regimens included: 5-FU/leucovorin/liposomal irinotecan (category 1 for metastatic disease), FOLFIRI, FOLFIRINOX, 5-FU/leucovorin/oxaliplatin (OFF), FOLFOX, CapeOx, capecitabine, and continuous infusion 5-FU (**Table 1**) (8–13). However, the use of 5-FU/leucovorin in second-line therapy may not achieve better clinical results. In the PANCREOX phase III clinical study, 108 patients with advanced pancreatic cancer who received gemcitabine as first-line treatment were randomized to receive second-line mFOLFIRINOX or 5-FU/leucovorin. The mPFS was 3.1 months and 2.9 months, and the mOS was 6.1 months and 9.9 months (9). Therefore, it is imperative to explore other second-line treatment options that may provide better survival benefits for patients with pancreatic cancer.

**Table 1:** The results of clinical studies of NCCN-recommended second-line treatment options for pancreatic cancer (median progression-free survival and median overall survival).

Study name	Phase	Reference	Regimen	mPFS	mOS
CONKO-003	III	H. Oettle et al. (8)	fluorouracil/leucovorin/oxaliplatin	2.9 months	5.9 months
PANCREOX	III	Sharlene Gill et al. (9)	fluorouracil/leucovorin/oxaliplatin	3.1 months	6.1 months
SWOG S1115	II	Vincent Chung et al. (10)	fluorouracil/leucovorin/oxaliplatin	2.0 months	6.7 months
NAPOLI-1	III	Andrea Wang-Gillam et al. (11)	fluorouracil/leucovorin/nanoliposomal irinotecan	3.1months	6.2 months
–	II	C Yoo al et (12).	fluorouracil/leucovorin/irinotecan	8.3 weeks	16.6 weeks
GISCAD	II	Alberto Zaniboni al et (13).	fluorouracil/leucovorin/irinotecan	3.2 months	5 months

TS-1 is a new generation of 5-FU oral compound preparation. Clinical studies have demonstrated that the efficacy of the TS-1 monotherapy in locally advanced or metastatic pancreatic cancer is superior to that of gemcitabine. It is an oral medication that is easy to use and well tolerated in clinical practice. In GEST Phase III clinical trial study, TS-1 (S-1) is no inferior to gemcitabine (GEM) in terms of OS in treatments of patients with metastatic pancreatic cancer or locally advanced pancreatic cancer. The mOS was 9.7 months for S-1 and 8.8 months for GEM (4). In the Japanese JASPAC-01 phase III clinical trial for comparing the adjuvant chemotherapy effect of S-1 and GEM after radical resection of pancreatic cancer, the chemotherapy effect of S-1 was significantly better than that of GEM, with a 5-year survival rate of 24.4% for GEM and 44.1% for S-1 (14). Since 2018, CSCO guidelines for pancreatic cancer have listed TS-1 monotherapy as the first-line chemotherapy method for metastatic pancreatic cancer. This patient also achieved PR (partial response) during initial treatment with TS-1 chemotherapy, and the efficacy lasted more than 6 months before first-line treatment failure, therefore, we still considered TS-1 in second-line medication.

The special microenvironment-dense stroma of pancreatic cancer affects drug presentation, which seriously restricts the efficacy of chemotherapy. In recent years, more and more clinical studies show that therapeutic strategies targeting the pancreatic cancer microenvironment combined with chemotherapy have unique therapeutic advantages. Among the regimens, although cetuximab in combination with gemcitabine and oxaliplatin for treatment of metastatic pancreatic cancer does not increase response and survival in patients with metastatic pancreatic cancer, the overall response was 33% and 31% of patients obtained stable condition (15). A phase III clinical trial showed that for locally advanced or metastatic pancreatic cancer, erlotinib in combination with gemcitabine showed an efficacy of statistical significance compared with gemcitabine in both OS and PFS. Despite this regimen preliminarily reflected some treatment advantages of small molecule TKI drugs combined with chemotherapeutic drugs, the OS achieved only increased by 9 days (6.24 months vs 5.91 months; P = 0.038), and the PFS was only prolonged by 6 days (3.75 months vs 3.55 months, P = 0.004) (16). Since the combination of erlotinib and gemcitabine, compared with gemcitabine monotherapy, has some limitations in prolonging OS and PFS in patients with locally advanced or metastatic pancreatic cancer, we need to explore a new small molecule TKI drug combined with chemotherapy for the treatment of pancreatic cancer.

Anlotinib, is a newly developed oral small molecule reverse transcriptase inhibitor. Compared with other RTK inhibitors such as sorafenib, sunitinib, and pazopanib, anlotinib can inhibit multiple targets such as VEGFR, PDGFR, FGFR, and c-Kit. In ALTER0302 (17) and ALTER-0303 (18), anlotinib improved progression-free survival (PFS) and overall survival (OS) in patients with advanced NSCLC who have failed second-line therapy. Other studies have shown that anlotinib is also effective in treatment of soft tissue sarcomas and medullary thyroid carcinomas (19) (20). Therefore, anlotinib may play a therapeutic role in a variety of malignancies. Zhang et al. revealed that anlotinib inhibited pancreatic cancer cell proliferation and induced its apoptosis (21). Yang et al. found that anlotinib had a killing effect on pancreatic cancer cells both *in vivo* and *in vitro* (22). Hence, anlotinib is expected to achieve a therapeutic advantage in the treatment of locally advanced and metastatic pancreatic cancer.

A retrospective study of anlotinib plus AG regimen in patients with advanced pancreatic cancer was reported at the poster of American Society of Clinical Oncology Gastrointestinal (ASCO GI) Cancers Symposium 2022. The results showed that the mPFS was 5.0 months and mOS was 9.0 months in the anlotinib combination group, which were significantly improved compared with those of AG regimen (mPFS=2.7 months; mOS=6.0 months) (23).

Based on the above studies demonstrating the potential advantages of TS-1 and anlotinib in the treatment of pancreatic cancer, we chose to combine these two drugs as the second-line regimen for this patient. After 2.5 months of second-line treatment, the tumor tended to shrink. During subsequent treatment and secondary radiotherapy, the condition remained stable without severe and uncontrollable adverse effects. To date, this patient has achieved a progression-free survival of 14 months and continues to remain progression-free. When treated with TS-1 in combination with anlotinib, the survival benefit that the patient obtained exceeded that shown in clinical studies related to first-line and second-line regimens recommended by guidelines. Therefore, we concluded that this patient achieved a good outcome when treated with TS-1 in combination with anlotinib in the second-line phase.

The therapeutic advantage of anlotinib for pancreatic cancer may lie in its effect on targeting the tumor microenvironment: ①Pancreatic stellate cells (PSCs), a major component of the tumor microenvironment (TME) of pancreatic cancer, expel a large number of extracellular matrix components, such as laminin, collagen, and fibronectin (24), inhibit the absorption of chemotherapeutic drugs and trigger drug resistance (25). ②Another major component of TME in pancreatic cancer, cancer-associated fibroblasts (CAFs), may enhance resistance to chemotherapy by promoting tumor fibrosis (26) (27). ③Finally, the pancreatic cancer microenvironment also has the characteristics of tumor vascular hypo-perfusion. The inefficient drug delivery caused by it may be an important factor leading to chemo-resistance in pancreatic cancer. Hence, the hemo-perfusion that alters the tumor microenvironment has the potential to improve the efficacy of chemotherapeutic drugs (28). The targets of action of anlotinib include VEGFR2/3, PDGFRα/β associated with tumor vascular survival ability (VSA), and FGFR1-4 associated with fibrosis (29). We hypothesize that anlotinib may synergistically improve the efficacy of chemotherapeutic agents by improving the hemoperfusion of the tumor microenvironment of pancreatic cancer and reducing the degree of fibrosis. Therefore, our center also initiated the clinical study of anlotinib targeted therapy combined with TS-1 chemotherapy for advanced pancreatic cancer (trial ethics number: ChiCTR2000032669) in order to solve the treatment bottleneck of such patients and improve the prognosis of patients with pancreatic cancer.

All adverse events (AEs) appeared to be manageable in the phase I trial. The overall incidence of AE with anlotinib was 100%, while 29% of patients reported Grade 3/4 AEs, including hand-foot skin reaction (5%), hypertension (10%), triglyceride elevation (10%), and lipase elevation (5%) (30). However, it also should be noted that patients receiving anlotinib treatment had a high occurrence of triglyceride and cholesterol elevation. Although these implications did not induce noticeable symptoms; such events were significantly more common in patients treated with anti-VEGFR TKIs (31). The 2022 ASCO GI Abstract 556 report mentioned that the rate of hematological adverse reactions in the anlotinib + chemotherapy group was not significantly higher than that in the chemotherapy monotherapy group, and some non-hematological toxicities in the anlotinib + chemotherapy group, such as hypertension, hand-foot syndrome, diarrhea, etc., may be related to anlotinib. In this case, the patient developed first-degree hypertension and Grade I hand-foot syndrome, both of which were well monitored (23).

Since the chemotherapy combined with targeted therapy reflected unique therapeutic advantages, including more convenient administration method (both TS-1 and anlotinib were administered orally), reduction of hospitalization times, and economic burden of patients, we finally selected the regimen of anlotinib targeted therapy combined with TS-1 chemotherapy for the treatment, by which good efficacy was obtained.

## Data Availability Statement

The original contributions presented in the study are included in the article/supplementary material. Further inquiries can be directed to the corresponding authors.

## Ethics Statement

The studies involving human participants were reviewed and approved by Ethics Committee of Guangxi Medical University Cancer Hospital. The patients/participants provided their written informed consent to participate in this study. Written informed consent was obtained from the individual(s) for the publication of any potentially identifiable images or data included in this article.

## Author Contributions

Conception/design: XL. Provision of study materials or patients:DL, SL, and QL. Collection and/or assembly of data: XL, DL, YZL, and JW. Data analysis and interpretation: XL and YQL. Manuscript writing: DL and XL. All authors contributed to the article and approved the submitted version.

## Funding

The Guangxi Medical and Health Appropriate Technology Development and Application Project (Grant No: S2020100). The Self-Raised Funds of Guangxi Health Department (grant no. Z20210852 and grant no. Z20190102). National Natural Science Foundation of China, Regional Fund Project (Grant No: 82060545). Guangxi Natural Science Foundation of China jointly-funded project, (Grant No: 2019GXNSFAA245073).

## Conflict of Interest

The authors declare that the research was conducted in the absence of any commercial or financial relationships that could be construed as a potential conflict of interest.

## Publisher’s Note

All claims expressed in this article are solely those of the authors and do not necessarily represent those of their affiliated organizations, or those of the publisher, the editors and the reviewers. Any product that may be evaluated in this article, or claim that may be made by its manufacturer, is not guaranteed or endorsed by the publisher.
